# Invitation to the 15^th^ Annual Meeting of the European Hair Research Society, Jerusalem, Israel, July 6–9, 2011

**DOI:** 10.4103/0974-7753.77509

**Published:** 2010

**Authors:** Abraham Zlotogorski

**Affiliations:** Department of Dermatology, Hadassah - Hebrew University Medical Center, Jerusalem, Israel. E-mail: jerusalem2011@ehrs.org

The rapid and evolving nature of hair research has led to the foundation of the European Hair Research Society (EHRS) in 1989, dedicated to the advancement of basic and clinical research focused on hair. Since the first meeting in Brussels, Belgium, the Society has held annual meetings in different cities in Europe, which attract more and more hair experts from universities, research institutions and industries from all over the world. Indeed, the EHRS stands in close contact with international hair research societies, and together with the North American Hair Research Society, the Korean Hair Research Society, the Society for Hair Science Research (Japan), the Australasian Hair and Wool Research Society, and the Indian Hair Research Society, co-organizes international scientific meetings.

**Figure 1:**
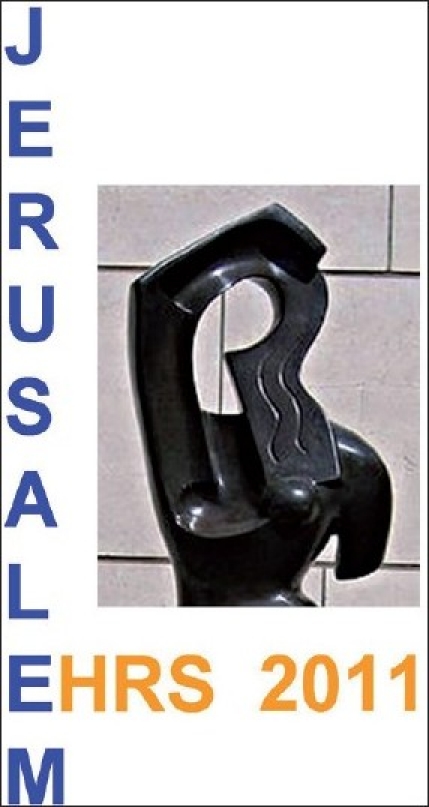
EHRS 2011, Jerusalem logo

The 15^th^ annual meeting of the EHRS will be held this year in Jerusalem, Israel, from July 6–9, 2011 [Figure 1]. The scientific program promises to be especially intriguing, and will include special lectures from leading scientists (please follow updates on our website http://www.ehrs.org or go directly to http://www.ehrs.org/conference/2011jerusalem/). The main conference will be preceded by a specialized hair course covering aspects of hair anatomy, pathology and genetics, fungal and parasitic infestations of the scalp, drugs affecting the hair and hair-associated contact dermatitis. The course will also present the basic and updated methods of hair trichoscopy. The opening lecture will be exceptionally attractive, and will discuss the momentous topic of “Hair in Movies”.

During the following two days, all major aspects of basic and clinical hair research will be discussed, from hair follicle physiology and biochemistry and hair growth cycles, through various pathological conditions of hypotrichosis and hirsutism, alopecia areata, androgenetic and cicatricial alopecia to various techniques of hair imaging and medical, molecular, cosmetic and surgical methods for hair regrowth or removal.

Prof. Desmond Tobin was selected to give the prestigious John Ebling lecture, an honor awarded to leading scientists in hair research for their exceptional achievements in this field. The topic of the lecture this year is the hair follicle melanocyte. In addition to plenary lectures on current topics of broad interest, short talks will be selected from the submitted abstracts and will be presented. We expect a large poster session, incorporating topics from all fields of hair research. The best posters and lectures will be honored with a Poster and Lecture Prizes. The EHRS is dedicated to the encouragement of the scientific careers of young investigators, and also grants the annual Jurgen Schweizer Prize for the best presentation during the meeting. Additionally, a Travel Grant will be provided to selected delegates after acceptance of their abstract. But above all, the EHRS allows young people to interact with leading hair researchers in an unhurried, intimate atmosphere. The unperturbed atmosphere of the EHRS meetings allows also for informal close interactions, and these unscripted meetings make the EHRS especially valuable and distinctive.

The venue of the EHRS 2011 will be the Ramada Hotel, located close to the heart of the city of Jerusalem. In addition to enjoying the scientific spirit of this meeting, delegates will also have the opportunity to appreciate the beauties of Jerusalem and its surroundings. Jerusalem is unique for incorporating a magnificent mix of cultures and people, and its breathtaking historical sights are sure to become an unforgettable experience for visitors. The social itinerary will include a visit to the Israel Museum, Light and Sound Show at the David Tower, Tour of the Old City of Jerusalem, Masada and the Dead Sea.

We invite you to submit abstracts for posters and oral presentations via jerusalem2011@ehrs.org. Early registration via jerusalem2011@ehrs.org by April 15^th^. We look forward to welcoming you in Jerusalem on 6–9 July, 2011.

Yours friendly,



